# Longitudinal Assessment of an ELISPOT Test for Mycobacterium tuberculosis Infection

**DOI:** 10.1371/journal.pmed.0040192

**Published:** 2007-06-12

**Authors:** Philip C Hill, Roger H Brookes, Annette Fox, Dolly Jackson-Sillah, David J Jeffries, Moses D Lugos, Simon A Donkor, Ifedayo M Adetifa, Bouke C de Jong, Alex M Aiken, Richard A Adegbola, Keith P McAdam

**Affiliations:** Bacterial Diseases Programme, Medical Research Council Laboratories, Banjul, The Gambia; University of California San Francisco, United States of America

## Abstract

**Background:**

Very little longitudinal information is available regarding the performance of T cell-based tests for Mycobacterium tuberculosis infection. To address this deficiency, we conducted a longitudinal assessment of the enzyme-linked immunosorbent spot test (ELISPOT) test in comparison to the standard tuberculin skin test (TST).

**Methods and Findings:**

In tuberculosis (TB) contacts we repeated ELISPOT tests 3 mo (*n* = 341) and 18 mo (*n* = 210) after recruitment and TSTs at 18 mo (*n* = 130). We evaluated factors for association with conversion and reversion and investigated suspected cases of TB. Of 207 ELISPOT-negative contacts, 51 (24.6%) had 3-mo ELISPOT conversion, which was associated with a positive recruitment TST (odds ratio [OR] 2.2, 95% confidence interval [CI] 1.0–5.0, *p* = 0.048) and negatively associated with bacillus Calmette-Guérin (BCG) vaccination (OR 0.5, 95% CI 0.2–1.0, *p* = 0.06). Of 134 contacts, 54 (40.2%) underwent 3-mo ELISPOT reversion, which was less likely in those with a positive recruitment TST (OR 0.3, 95% CI 0.1–0.8, *p* = 0.014). Between 3 and 18 mo, 35/132 (26.5%) contacts underwent ELISPOT conversion and 28/78 (35.9%) underwent ELISPOT reversion. Of the 210 contacts with complete results, 73 (34.8%) were ELISPOT negative at all three time points; 36 (17.1%) were positive at all three time points. Between recruitment and 18 mo, 20 (27%) contacts had ELISPOT conversion; 37 (50%) had TST conversion, which was associated with a positive recruitment ELISPOT (OR 7.2, 95% CI 1.4–37.1, *p* = 0.019); 18 (32.7%) underwent ELISPOT reversion; and five (8.9%) underwent TST reversion. Results in 13 contacts diagnosed as having TB were mixed, but suggested higher TST sensitivity.

**Conclusions:**

Both ELISPOT conversion and reversion occur after M. tuberculosis exposure. Rapid ELISPOT reversion may reflect M. tuberculosis clearance or transition into dormancy and may contribute to the relatively low reported ELISPOT conversion rate. Therefore, a negative ELISPOT test for M. tuberculosis infection should be interpreted with caution.

## Abstract 2

Philip Hill and colleagues report that both ELISPOT conversion and reversion occur after*M. tuberculosis* exposure in an endemic country and that the ELISPOT results agree poorly with results from the tuberculin skin test.

## Editors' Summary

**Background.:**

Tuberculosis is a contagious bacterial infection, usually of the lungs. People with active tuberculosis spread the causative bacterium *(Mycobacterium tuberculosis)* in airborne droplets whenever they cough or sneeze. Most people exposed to M. tuberculosis in this way never become ill—their immune system successfully contains the infection. However, the bacteria remain dormant in the body and can cause disease years later if host immunity declines because of, for example, infection with the human immunodeficiency virus (HIV). Consequently, to control the spread of tuberculosis, individuals who have been in contact with people with active tuberculosis need to be tested for infection with M. tuberculosis and treated with antituberculosis drugs if positive. The standard test for infection is the tuberculin skin test (TST). In this, bacterial antigens (proteins that the immune system recognize as foreign) are injected under the skin. The immune system of infected individuals attacks the antigen and produces a hard swelling at the injection site. Unfortunately, this test does not detect all M. tuberculosis infections and an alternative, laboratory-based test has recently been developed. During M. tuberculosis infections, immune system cells called T lymphocytes produce interferon gamma. This protein activates macrophages, immune system cells that kill bacteria. The ELISPOT (enzyme-linked immunosorbent spot) test measures interferon gamma production by T lymphocytes.

**Why Was This Study Done?:**

Commercial ELISPOT tests are available for the diagnosis of M. tuberculosis infection, but little is known about how they perform when used in repeat tests in individuals or whether the TST or ELISPOT test is better at predicting later development of tuberculosis. In this study, the researchers investigated these questions in a longitudinal assessment of the ELISPOT test in Gambians exposed to active tuberculosis.

**What Did the Researchers Do and Find?:**

The researchers recruited people who had been in contact with active tuberculosis, did ELISPOT tests and TSTs at recruitment, then repeated the ELISPOT test after three months and both tests in some participants after 18 months. They analyzed how often ELISPOT conversion (a change from a negative to a positive result indicating the development of an active immune response) and reversion (a change from a positive to a negative result reflecting clearance of the bacteria or its entry into a dormant state) occurred, whether the TST results mirrored these changes, and which characteristics of the participants were associated with conversion or reversion. A quarter of participants who initially had a negative ELISPOT result had a positive result at three months, a conversion that was associated with a positive TST at recruitment. ELISPOT reversion at three months, by contrast, was associated with an initially negative TST and occurred in nearly half the participants. However, about a third of the participants had negative ELISPOT results at all three time points and a fifth had positive results at all times. Overall, the two tests agreed in 73% and 60% of the participants at recruitment and at 18 months, respectively. Finally, among the 13 contacts who developed active tuberculosis, some were initially positive in both tests but others showed subsequent conversion in one, both or neither test.

**What Do These Findings Mean?:**

These findings indicate that both ELISPOT conversion and reversion occur after initial screening for M. tuberculosis infection. In addition, they suggest that the immune system responses to M. tuberculosis detected by TST and the ELISPOT test occur over different time scales and so the two tests might differ in their ability to detect M. tuberculosis infections at different times after exposure to the bacteria. Because very few contacts developed active tuberculosis, the findings do not indicate which test best predicts disease development after M. tuberculosis infection. Further studies are needed to provide this information and to unravel the complexities of ELISPOT conversion and reversion after exposure to M. tuberculosis. Importantly, however, the high frequency of ELISPOT reversion seen in this study suggests that a negative ELISPOT result may not reflect a lack of infection after exposure to M. tuberculosis and must, therefore, be interpreted with caution.

**Additional Information.:**

Please access these Web sites via the online version of this summary at http://dx.doi.org/10.1371/journal.pmed.0040192.

- The US Centers for Disease Control and Prevention provide fact sheets from the Division of Tuberculosis Elimination about tuberculosis, its testing and diagnosis, and its treatment
- MedlinePlus Encyclopedia contains information on tuberculosis and the tuberculin skin test (in English and Spanish)
- The American Lung Association offers fact sheets on tuberculosis and on the tuberculin skin test

## Introduction

Recent work suggests that a T cell-based assay for interferon gamma, the enzyme-linked immunosorbent spot test (ELISPOT), has promise in the diagnosis of Mycobacterium tuberculosis infection after exposure to a known tuberculosis (TB) patient [1–3]. However, commercialisation of two T cell-based tests for the diagnosis of M. tuberculosis infection (T-Spot.TB by Oxford Immunotec and Quantiferon-TB Gold by Cellestis) preceded substantial longitudinal assessment of either test. Apart from two small studies [4,5], longitudinal assessment of an ELISPOT assay for M. tuberculosis infection has been confined to studies of TB patients undergoing treatment. These studies have consistently shown that significant ELISPOT reversion occurs over a treatment course [6–9]. One longitudinal assessment of the Quantiferon test with respect to M. tuberculosis infection has been performed, in India [10].

Longitudinal assessment of T cell-based tests for M. tuberculosis infection should comprise documentation of the conversion rate in individuals initially testing negative after M. tuberculosis exposure, the reversion rate in those initially testing positive, assessment of factors for association with each phenomenon, and the relationship between an initial test result and the future development of disease. A comparison with the traditional tuberculin skin test (TST) would be optimal. Therefore we established a large cohort of consecutively recruited TB patient contacts, conducted repeated ELISPOT and TSTs, and investigated those suspected of developing TB disease.

## Materials and Methods

### Participants

TB patients over 15 years of age were recruited consecutively from the major government TB clinic in Banjul, The Gambia, and from the Medical Research Council outpatient's clinic, as previously described [2]. Included patients had two sputum smear samples positive for acid-fast bacilli and with M. tuberculosis isolated upon culture. Household contacts of the TB patients were eligible for inclusion in the study if they were at least 15 years of age. Contacts were interviewed, examined, and a blood sample taken for ELISPOT and HIV test. Immediately afterwards they underwent a TST (2 Tuberculin Units [TU], PPD RT23, Statens Serum Institut, http://www.ssi.dk). They were asked to have a repeat ELISPOT test at 3 and 18 mo. A “subcohort” of 196 consecutively recruited contacts, with ELISPOT and TST results from recruitment, were asked to have a repeat TST at 18 mo as well. We did not conduct “two-step” TSTs at either recruitment or follow-up.

TST conversion was defined as a positive test (≥10 mm induration) plus an increase in induration of at least 6 mm [11]. All TST converters were asked to have a chest x-ray and a clinical examination. Those able to produce sputum underwent sputum analysis. Those diagnosed with TB disease were referred to the National Programme for free treatment and excluded from further longitudinal analyses. There is no current practice of preventive anti-TB treatment in The Gambia. HIV-positive individuals were referred for consideration for free antiretroviral treatment.

The study was approved by The Gambia Government/Medical Research Council joint Ethics Committee. Written informed consent was obtained from all study participants.

### Laboratory Procedures

Sputum smears were prepared and stained with auramine-phenol [12] and confirmed by Ziehl-Neelsen. Decontaminated specimens were inoculated into Lowenstein-Jensen medium and BACTEC 9000 MB liquid medium for isolation and identification of *M. tuberculosis,* as previously described [13]. Testing for HIV-1 or HIV-2 infection was by competitive ELISA (Wellcome Laboratories, Dartford, Kent, UK) and Western blot (Diagnostics Pasteur, http://www.sanofipasteur.com), as previously described [14].

The ex-vivo ELISPOT assays for IFNγ were performed on fresh samples onsite as previously described [15]. Pooled sequential 15-mer peptides, overlapping by 10 amino-acid residues, of ESAT-6 and CFP-10 proteins (Advanced Biotechnology Centre, www.imperial.ac.uk/advancedbiotechnologycentre) were used as stimulatory antigens at 5 μg/ml. The positive control was phytohaemagglutinin (Sigma-Aldrich, http://www.sigmaaldrich.com). All antigens were tested in duplicate wells. Assays were scored by an ELISPOT counter (AID-GmbH, http://www.aid-diagnostika.com). Positive test wells were predefined as containing at least 8 spot-forming units (SFU) more than negative control wells [16]. For a positive ESAT-6/CFP-10 result it was necessary for at least one of the two pools of overlapping peptides to be positive. Phytohaemagglutinin wells were set to at least 150 SFU/well/2 × 10^5^ above negative control wells. Negative control wells were required to have less than 20 SFU. These criteria are the same as those we have previously documented and are more stringent than those recommended for the commercial T-spot assay [3]. ELISPOT conversion and reversion was defined as a newly positive test or negative test respectively, plus a change in the combined ESAT-6 and CFP-10 count (above the negative control) of at least 6 SFU/well/2 × 10^5^ (30 SFU/million cells). Laboratory staff were blinded as to the characteristics of the individuals tested.

For molecular subtyping of index case isolates, we extracted mycobacterial DNA using CTAB and chloroform, as previously described [17], and assessed its concentration and purity by spectrophotometry. We performed spoligotyping using membranes from Isogen Biosciences (http://www.isogen-lifescience.com), as previously described [18] and analysed the results with software that we designed using Matlab software (MathWorks, http://www.mathworks.com).

### Data Management and Statistical Analysis

The number of SFU in each ELISPOT well were automatically entered into a database. All other data were entered using double data entry into an ACCESS database and checked for errors. Agreement between the qualitative test results was assessed by the kappa statistic and the significance of the discordance was assessed by McNemar test. Random effects logistic regression models, taking into account household clustering, were used to separately assess the relationship between exposure and test conversion and reversion between recruitment and 3 mo, 3 mo and 18 mo, and recruitment and 18 mo. All statistical analyses were conducted using Stata software (version 8; Stata Corp, http://www.stata.com).

## Results

### Recruitment Results

**Figure 1:**
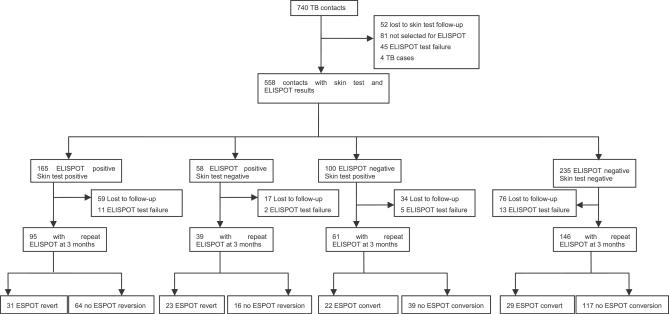
Study Profile: Recruitment and 3-Mo ELISPOT Conversion and Reversion

We recruited 740 contacts of 177 TB patients (Figure 1). Four were identified as coprevalent TB patients, and 558 were selected for ELISPOT and had TST and ELISPOT results: 165 (29.6%) were ELISPOT positive and TST positive, 58 (10.4%) were ELISPOT positive and TST negative, 100 (17.9%) were ELISPOT negative and TST positive, and 235 (42.1%) were negative by both tests. The agreement between the two tests was 73% (kappa = 0.43) and there was significant discordance identified (McNemar test: *p* < 0.001). Of 554 contacts tested, 15 (2.7%) were HIV positive. Since HIV positivity was not significantly associated with conversion or reversion of either test, such individuals were not excluded from the analysis. There were no significant differences in the age and sex characteristics of those contacts with results at recruitment, 3-mo follow-up, or 18-mo follow-up, compared to their source population.

### 3-Mo ELISPOT Results

Figure 1 shows the details of the 3-mo follow-up of the 558 contacts with results at recruitment. Of 341 contacts who had ELISPOT results at 3 mo, 133 (39%) were ELISPOT positive and 208 (61%) were ELISPOT negative. Of the initially negative individuals, 51 (24.6%) had ELISPOT conversion (Table 1), which was more likely in those who were TST test positive at recruitment (odds ratio [OR] 2.2, 95% confidence interval [CI] 1.0–5.0, *p* = 0.048) and was less likely in those with a BCG scar (OR 0.5, 95% CI 0.2–1.0, *p* = 0.060). Of the initially positive contacts, 54 (40.2%) underwent ELISPOT reversion, which was less common if the recruitment TST was positive (OR 0.3, 95% CI 0.1–0.8, *p* = 0.014); no other factors were found to be associated with ELISPOT reversion at 3 mo. The median increase in ELISPOT count for those with conversion was 21 SFU (range 7 to 192; interquartile range [IQR] 15 to 31 SFU). The median decrease in ELISPOT count for those undergoing reversion was 21 SFU (range 6 to 334; IQR 11 to 33 SFU).

**Table 1:**
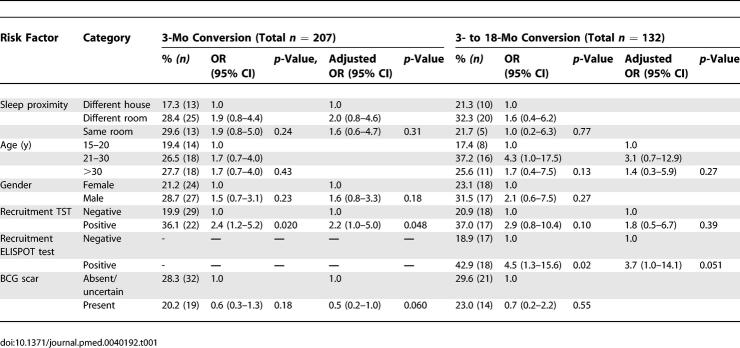
Evaluation of Possible Factors Associated with ELISPOT Test Conversion at 3 Mo and at 18 Mo in TB Patient Contacts

### ELISPOT Results between 3 and 18 Mo

**Figure 2:**
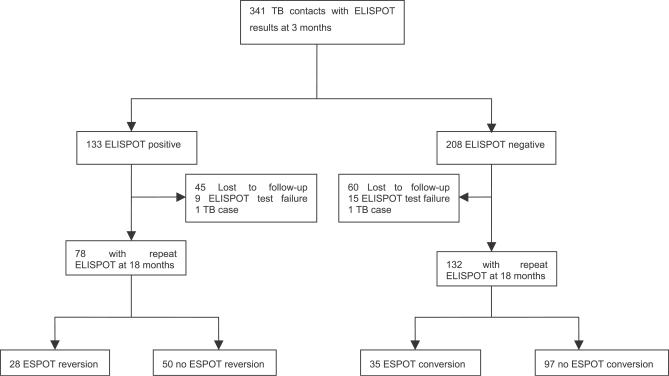
Study Profile: 3-Mo and 18-Mo ELISPOT Results

Figure 2 shows the details of the 18-mo follow-up of the 341 contacts with ELISPOT results at 3 mo. Two contacts became TB patients between 3 and 18 mo, and 210 (90%) of the 234 bled for ELISPOT at 18 mo had adequate results for analysis. Of these 210 contacts, 73 (34.8%) were ELISPOT negative at all three time points; 36 (17.1%) were positive at all three time points. Of contacts who were negative by ELISPOT at 3 mo, 35 (26.5%) underwent ELISPOT conversion at 18 mo. Such conversion was associated with having been initially positive by ELISPOT at recruitment, although this was of borderline significance (Table 1; adjusted OR 3.7, 95% CI 1.0–14.1, *p* = 0.051). Of contacts who were positive at 3 mo, 28 (35.9%) underwent ELISPOT reversion. ELISPOT reversion decreased with increasing age (Table 2; *p* = 0.010) and in those who had been ELISPOT positive at recruitment (Table 2; OR 0.2, 95% CI 0.05–0.8, *p* = 0.020). The median increase in ELISPOT count for those with conversion was 18 SFU (range 6 to 132; IQR 12 to 29 SFU). The median decrease in ELISPOT count for those undergoing reversion was 20 SFU (range 7 to 142; IQR 12 to 31 SFU).

**Table 2:**
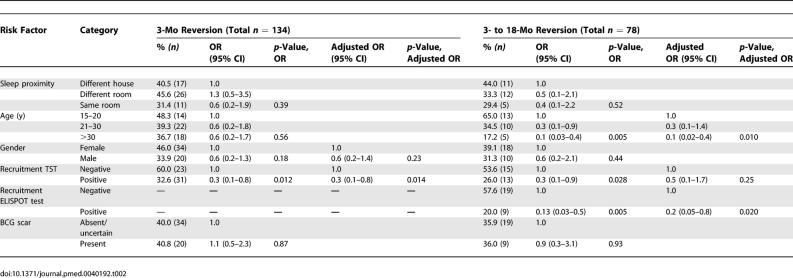
Evaluation of Possible Factors Associated with ELISPOT Test Reversion at 3 Mo and at 18 Mo in TB Patient Contacts

### ELISPOT and TST Results between Recruitment and 18 Mo

**Figure 3:**
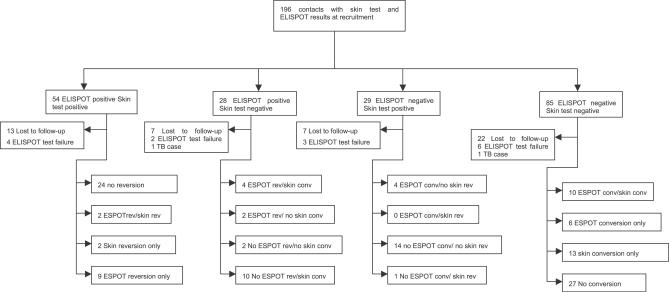
Study Profile: Recruitment and 18-Mo ELISPOT and TST Conversion and Reversion

Figure 3 shows the details of the follow-up of the 196 consecutively recruited contacts for repeat ELISPOT and TST at 18 mo. At 18 mo 45 (34.6%) of the contacts, with complete results at 18 mo, were ELISPOT positive and TST positive, nine (6.9%) were ELISPOT positive and TST negative, 43 (33.1%) were TST positive and ELISPOT negative, and 33 (25.4%) were negative by both tests. The agreement between the two tests was 60% (kappa = 0.25) and significant discordance was identified (McNemar test: *p* < 0.001).

Of the 75 contacts who were initially ELISPOT negative and had complete results at follow-up, 20 (27%) had ELISPOT conversion and none of the factors considered were associated (Table 3). Of the 74 contacts who were initially TST negative and had complete results at follow-up, 37 (50%) had TST conversion, which was more likely to occur in those who had been ELISPOT positive at recruitment (Table 3; OR 7.2, 95% CI 1.4–37.1, *p* = 0.019). There was little difference in agreement between ELISPOT and TST conversion, when two alternative definitions for conversion for each test were assessed against each other (Table 4). Of 55 ELISPOT-positive contacts, 18 (32.7%) underwent ELISPOT reversion. Of the 56 TST-positive contacts, only five (8.9%) underwent TST reversion. None of the factors considered were found to be associated with either ELISPOT or TST reversion (Table 5). The median increase in ELISPOT count for those with ELISPOT conversion was 18 SFU (range 6 to 132; IQR 11 to 33 SFU). The median increase in induration for those with TST conversion was 15mm (range 7 to 27; IQR 12 to 17 SFU). The median decrease in ELISPOT count for those with ELISPOT reversion was 19 SFU (range 7 to 340; IQR 12 to 31 SFU). The median decrease in induration for those with TST reversion was 14 mm (range 8 to 23 mm).

**Table 3:**
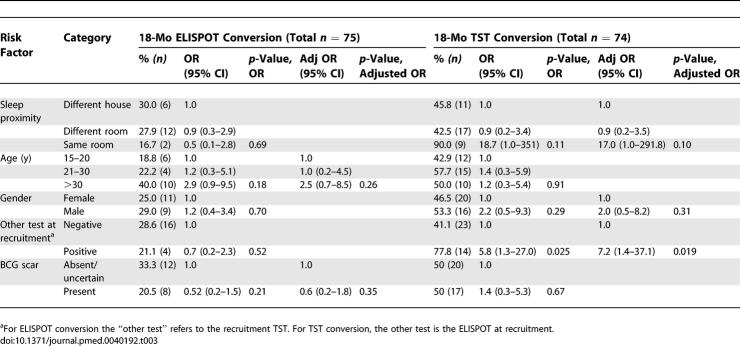
Evaluation of Possible Factors Associated with ELISPOT Test and TST Conversion after 18 Mo in TB Patient Contacts

**Table 4:**
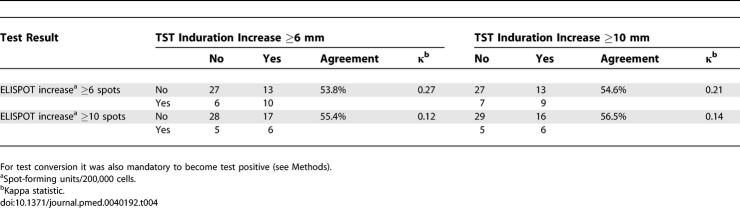
Conversion of the Tuberculin and ELISPOT Tests in Relation to Each Other, According to Different Cut-Offs in Those That Were Initially Negative by Both Tests (*n* = 56)

**Table 5:**
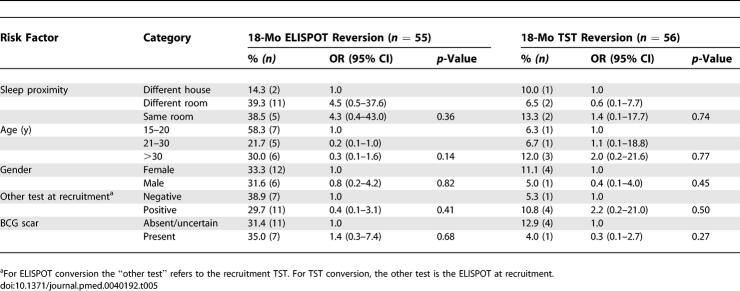
Evaluation of Possible Factors Associated with ELISPOT or TST Reversion after 18 Mo in TB Patient Contacts

Of the 56 contacts who were initially negative by both tests, 6/16 (37.5%) ELISPOT converters did not undergo TST conversion. All six had 0 mm of induration at both time points and their median ELISPOT count increase was 22 SFU (range 18 to 133 SFU). Similarly, 13/23 (56.5%) TST converters did not undergo ELISPOT conversion: their median recruitment ELISPOT count was 3 SFU (range 0 to 7 SFU) and their median change in count was 3 SFU (range −3 to 5 SFU). Ten contacts, initially negative by both tests, had conversion of both: the median increase in induration was 16mm (range 6 to 27 mm) and the median increase in ELISPOT count was 18 SFU (range 7 to 76 SFU). Five of the 130 contacts with TST and ELISPOT results at recruitment and 18 mo had TST conversion and ELISPOT reversion: the median increase in induration was 14 mm (range 10 to 19 mm), and the median drop in ELISPOT count was 19 SFU (range 9 to 25 SFU).

### Identification of Secondary TB Patients

Among 665 contacts with complete follow-up information regarding symptoms over the 18 mo period, 13 TB patients were identified: five at recruitment, four by 4 mo, two between 4 and 18 mo, and two after 18 mo (Table 6). All five diagnosed at recruitment were TST positive, and three were ELISPOT positive. Of the four individuals diagnosed by 4 mo, one had been negative by both ELISPOT and TST at recruitment and did not have TST conversion (no ELISPOT result at 3 mo), one was positive on both tests at recruitment, and two were positive on TST but negative by ELISPOT at recruitment; one of these underwent ELISPOT conversion. Of the two individuals diagnosed at 14 mo, one was negative by both tests at recruitment and 3 mo, the other was positive by TST at recruitment and underwent ELISPOT conversion at 3 mo. Of the two patients diagnosed after 18 mo, one was ELISPOT positive at recruitment and TST negative and later had TST conversion, the other was persistently ELISPOT negative but had TST conversion at 18 mo. All those with a negative ELISPOT test had a count of at least 3 SFU below the cut-off for a positive result. Six patients had both a cultured isolate and the isolate of their respective index case available; three had identical spoligotype patterns and three were different.

**Table 6:**
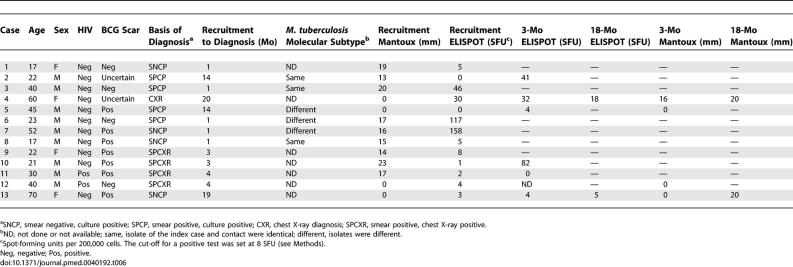
Characteristics of 13 TB Patients Diagnosed among TB Case Contacts

## Discussion

In this study of over 1,100 individual ELISPOT test results and nearly 800 TST results, we have shown that ELISPOT conversion and reversion occur after an initial post-M. tuberculosis exposure screening process. In contrast to the low rate of TST reversion over time, ELISPOT reversion occurred in 40% of ELISPOT-positive individuals at 3 mo, and in 36% of individuals between 3 and 18 mo. Conversely, the ELISPOT conversion rate was 27% at 18 mo compared to a TST conversion rate of 50% over the same time period. Conversion and reversion of the ELISPOT test are associated with identifiable risk factors that differ from those associated with TST conversion and reversion. These results provide new insights into the possible niche for ELISPOT in the diagnosis of latent M. tuberculosis infection and TB disease.

To our knowledge, only two small longitudinal studies of ELISPOT results in TB patient contacts have been reported. Ewer et al. [4] found a significant decline in ELISPOT counts at 18 mo in those who underwent prophylactic treatment, 11 untreated ELISPOT-negative adults had no significant change in count, while seven of 14 untreated ELISPOT-positive individuals underwent ELISPOT reversion; all seven had negative TST results at recruitment. The high reversion rate and association with a negative initial TST are consistent with the findings of our study. The lack of ELISPOT conversion can possibly be explained by the 4-mo time delay in conducting the initial ELISPOT screen. Wilkinson et al. [5] showed a significant early rise in the ELISPOT count in 33 Heaf test-positive individuals given isoniazid and rifampicin, then a reduction in count at 3 mo. There were no significant changes in counts in eight individuals who had opted for no treatment. A longitudinal assessment of the Quantiferon test conducted in India [10] included follow-up of 216 nursing and medical students. Overall, nine (24%) of 38 initially test-positive participants underwent test reversion. Consistent with our study, the investigators found that initially positive individuals were more likely to undergo Quantiferon test reversion if their initial TST was negative. The agreement between the TST and Quantiferon was higher at both time points than in our study. Furthermore, in contrast to our study, all those with TST conversion with at least a 10 mm increase in induration, had large increases in levels of IFN-γ on Quantiferon assay. It is important to note that there were several important differences between the Indian study and ours. For example, one TU was used for the skin test and individuals were more likely to have ongoing exposure to M. tuberculosis, being hospital based.

Conversion and reversion of the traditional TST following BCG vaccination have been reported in a TB-endemic tropical setting [19]. Although TST reversion would not be expected after only 3 mo [20], it is an important consideration when interpreting trends in positivity at a population level. It appears that TST reversion is greatest in the youngest age groups and after nontuberculous mycobacterial exposure, while the rate of TST conversion gradually increases with age [21–23]. At both ELISPOT follow-up time points, those who were in the youngest age group had the highest ELISPOT reversion rate in our study; this was statistically significant at the 18-mo time point. Therefore, it is important that a longitudinal assessment of the ELISPOT now be conducted in children.

That a large proportion of contacts undergo rapid reversion of the ELISPOT is consistent with the premise that ELISPOT responses are transient and generally require continued exposure to antigen to maintain high frequencies. While some reversion could reflect clearance of the organism, it may simply be a function of the M. tuberculosis life cycle, whereby the mycobacterium enters a dormant state in which it may not reliably secrete ESAT-6 and CFP-10, but preferentially secretes other antigens [24]. Using antigens preferentially secreted by M. tuberculosis in its dormant phase, it may prove possible to distinguish those who retain the infection from those who clear it [25]. It is quite possible, however, that ESAT-6 and CFP-10 are secreted intermittently by M. tuberculosis at all stages of its life cycle [26]. Furthermore, persistence of an “ELISPOT detectable” T cell response may occur in certain individuals in the absence of direct antigen stimulation [8]. That ELISPOT reversion between 3 and 18 mo was less likely in those that had previously been ELISPOT positive is of interest in this regard. Studies are underway in The Gambia to explore these issues.

ELISPOT and/or TST conversions have several possible explanations. First, the interval between initial exposure and TST conversion has been shown to be up to 6 wk after BCG vaccination [27] and 3–7 wk following known M. tuberculosis exposure [28]. The corresponding time interval for the ELISPOT test is not known. It is possible that it is shorter than that of the TST, as TST conversion at 18 mo was significantly more likely in those who had been ELISPOT positive at recruitment. However, ELISPOT conversion at 3 mo was slightly more likely in those who had been TST positive at recruitment. Such a mixed result is consistent with the secondary case data—one patient had been initially positive by ELISPOT and negative by TST, becoming positive by TST later; however for two patients the opposite was true. Second, it is likely that some contacts become exposed to M. tuberculosis for a period after their respective index case begins treatment. Third, it is possible for individuals to be exposed to an unrecognised case. This is supported by the fact that three of six “secondary” cases with molecular subtyping results had an isolate with a different spoligotype pattern than their respective index case. Finally, the TST is subject to boosting of waned immunity to tuberculous and nontuberculous past exposure. Therefore, the difference in conversion rates at 18 mo (27% for ELISPOT versus 50% for TST) may be due to a combination of factors: slightly increased TST sensitivity, different times to positivity, TST boosting, and early ELISPOT reversion. While 13 of 23 of TST converters did not undergo ELISPOT conversion, it is of note that only ten of 16 ELISPOT converters had TST conversion. It is therefore quite likely that certain individuals with genuine new M. tuberculosis infection preferentially respond to one test and not the other.

Our finding that ELISPOT conversion between 3 and 18 mo was associated with having been positive at recruitment adds further weight to the argument that ELISPOT reversion at 3 mo cannot be explained completely by clearance of the infection. As discussed above, one would expect intermittent antigen secretion to cause the ELISPOT test to “switch on,” “switch off,” and “switch on” again over time.

In this study we found that those with a visible BCG scar were less likely to undergo ELISPOT conversion. While this finding was of borderline statistical significance, it is consistent with the finding, by Soysal et al. [29], that TB patient contacts with a BCG scar in Turkey were less likely than those without to have a positive ELISPOT test. Soysal et al. argue that this is evidence in favour of the premise that BCG may protect against new M. tuberculosis infection.

In The Gambia we have used mathematical tools on results from over 1,000 individuals to identify a cut-off for positivity of the ELISPOT test [16] of eight spots per well (40 spots/million cells) above the negative control well when using two antigens, as opposed to five or six spots per well that has been used in some other studies [1]. The criterion we used for TST conversion was chosen because chance variation in the TST reading results had been reported to be less than 6 mm of induration in over 95% of individuals [11,30]. No criteria for ELISPOT conversion and reversion have been proposed. We consulted researchers experienced in working with ELISPOT assays in coming to these criteria and considered variability between duplicate test wells that we have previously reported [9]. It is also of note, in this regard, that there was little change in agreement between the and ELISPOT when we compared different criteria (Table 5). However, it is important that further detailed studies are conducted to clearly determine reproducibility of ELISPOT results.

There are other limitations of this study. First, 25% to 33% of the contacts were lost to follow-up between the different time points and a further 5% to 8% of contacts' samples were subject to test failure. While we did not find any significant differences in the basic characteristics of those followed versus those lost to follow-up, the study is vulnerable to unknown sources of bias. Second, in this cohort we did not have a TST and ELISPOT comparison at 3 mo. This comparison would be helpful with respect to understanding agreement between the two tests, although it is not ideal to have injection of mycobacterial antigens in between the other two time points. Third, while our secondary case information is useful, larger numbers will be required to draw definitive conclusions.

The results of this study have important implications for clinical practice and future research on T cell assays for M. tuberculosis infection. It is clear that ELISPOT reversion is much more frequent than TST reversion, and this key difference will be crucial in the interpretation of both tests in relation to each other, especially when there is no particular reference point in time for exposure to *M. tuberculosis.* Those using a T cell-based test to screen for M. tuberculosis infection should be cautious when interpreting a negative result. It is important that long-term follow-up of large numbers of case contacts be conducted to identify enough secondary cases to provide further insights into the meaning of rapid ELISPOT reversion and of discordant results. Such studies are underway in The Gambia. It will also be important to document the rates of ELISPOT conversion and reversion in children. These studies will help to define the ultimate niche for T cell-based tests in relation to TB.

## Abbreviations

BCG: bacillus Calmette-Guérin
CI: confidence interval
ELISPOT: enzyme-linked immunosorbent spot test
IQR: interquartile range
OR: odds ratio
SFU: spot-forming unit(s)
TB: tuberculosis
TST: tuberculin skin test
